# Factors for starting biosimilar TNF inhibitors in patients with rheumatic diseases in the real world

**DOI:** 10.1371/journal.pone.0227960

**Published:** 2020-01-24

**Authors:** Yoon-Kyoung Sung, Sun-Young Jung, Hyoungyoung Kim, Seongmi Choi, Seul Gi Im, Yu Sang Lee, Eun Jin Jang, Soo-Kyung Cho

**Affiliations:** 1 Department of Rheumatology, Hanyang University Hospital for Rheumatic Diseases, Seoul, Republic of Korea; 2 College of Pharmacy, Chung-Ang University, Seoul, Republic of Korea; 3 Department of Statistics, Kyungpook National University, Buk-gu, Daegu, Republic of Korea; 4 Department of Information Statistics, Andong National University, Andong, Republic of Korea; Brigham and Women’s Hospital, UNITED STATES

## Abstract

**Background:**

To identify factors for starting biosimilar TNF inhibitors (TNFI) in patients with rheumatic diseases.

**Methods and finding:**

Using a national claims database, we identified patients with rheumatoid arthritis (RA) or ankylosing spondylitis (AS) who had used TNFIs since they were approved in Korea in 2004. We assessed changes in the proportion of each form of TNFI used between 2004 and 2017. We then selected patients starting on TNFIs between 2013 and 2017 to identify factors for starting biosimilars. In RA (n = 4,216), biosimilars were more likely to be initiated in clinics [odds ratio (OR) 2.54] and in the metropolitan area (OR, 2.02), but were less likely to be initiated in general hospitals (OR 0.40) or orthopedics (OR 0.44). In AS (n = 2,338), biosimilars were common at the hospital level (OR 2.20) and tended to increase over the years (OR 1.16), but were initiated less in orthopedics (OR 0.07). In addition, RA patients were more likely to initiate biosimilars in combination with methotrexate (OR 1.37), but biosimilars were not initiated frequently by patients with higher comorbidity scores (OR 0.97) or receiving glucocorticoids (OR 0.67). The patient factors favoring biosimilar in AS use were not clear.

**Conclusions:**

In Korea, the proportion of biosimilar TNFIs has increased. Type of institution and physician specialty are more important than patient factors in affecting biosimilar use. In RA, biosimilar TNFIs tend to be initiated in combination with MTX, and are less likely to be initiated in patients taking glucocorticoids or in those with high comorbidities.

## Introduction

The introduction of biosimilars is likely to widen access and reduce treatment inequalities in inflammatory arthritis due to their lower cost compared to the originator biologics [1, 2]. In clinical trials, biosimilars showed equivalent efficacy and comparable safety to the originator products in the short term: 24 and 30 weeks, and no greater immunogenicity [3–5]. Therefore, biosimilars were approved for treating several rheumatic diseases by the European Medicines Agency (EMA) in 2013, and the US Food and Drug Administration (FDA) in 2016. Moreover, the recently updated European League Against Rheumatism (EULAR) recommendations for the management of rheumatoid arthritis (RA) recommend that biosimilars be considered as equivalent to their originators [6].

However, patients included in clinical trials differ from patients treated in routine care, who are often older and have more comorbidities or atypical disease presentations [7]. For that reason, observational studies have been performed to evaluate the effectiveness and safety of biosimilars in clinical practice, and these have also shown similar effectiveness and safety [8–12]. In spite of these encouraging results, several concerns with biosimilars still exist from the patients’ perspective in the real world [13, 14]. Patients’ lack of knowledge about biosimilars and concerns about their efficacy and safety may be related to the low adherence to these drugs. In addition, a nocebo effect, which causes adverse events owing to the negative expectations of patients, has been emphasized in biosimilar users with rheumatic diseases [15, 16].

Recent data in the DANBIO registry showed that switch outcomes in routine care were influenced by patient-related factors and non-specific drug effects, although one-year treatment retention rates were higher in switchers than in non-switchers. Such observational studies allow us to explore outcomes in unselected patient cohorts representing the whole disease spectrum, and may also provide insight into how biosimilars are employed and how they perform in the real world [12]. However, Danish national guidelines have stated that all patients with inflammatory arthritis treated with an originator must switch to a biosimilar for economic reasons [12]. This may make it difficult to identify factors encouraging the choice of biosimilars rather than originators in the real world.

In Korea, biosimilars were already approved in 2012. In addition, the reimbursement guidelines for patients with rheumatic diseases recommend biosimilars as equal to their originators, and this has led in clinical practice to the choice between them being made by patients and physicians. Therefore, this study aimed to identify factors favoring starting biosimilars of TNF inhibitors (TNFIs) in patients with rheumatic diseases in the real world when both kinds of drug are available.

## Methods

### Data source

All South Koreans are eligible for coverage under the National Health Insurance Program. A total of 50 million individuals, or virtually the entire population, are included in the Korean National Health Insurance Service (NHIS) database [17]. The database contains individual beneficiary information, in addition to healthcare service information such as diagnosis, procedures, prescriptions, and tests. We used data for Jan. 2002 to Dec. 2017 extracted from the NHIS by stratified sampling based on age, gender, and the diagnostic codes for RA and ankylosing spondylitis (AS). Our data encompass 50% of the total number of biologic disease-modifying antirheumatic drug (DMARD) users in Korea in the form of patients with RA or AS. It was not appropriate or possible to involve patients or the public in this work.

### Study population and design

#### Prevalence of the use of each TNFI

To evaluate the prevalence of the use of each TNFI, we identified patients with at least one prescription for TNFI for RA or AS since approval of TNFIs in Korea in 2004. We assessed the proportions of each TNFI used by all TNFI users between 2004 and 2017. The dates of approval of each drug are given in S1 Table.

#### Identification of first users of TNFIs

To identify factors for starting biosimilar TNFIs in RA or AS, we located new starters of TNFIs in several steps. First, we selected definite RA or AS patients by excluding patients who were ever assigned diagnostic codes for both RA and AS. Second, we selected patients who used TNFIs between 2013 and 2017 because the first biosimilar TNFIs were approved in Korea in November 2012 (RA) and December 2012 (AS). Then we identified new users of TNFIs between 2013 and 2017. The first TNFI for a given patient was defined as the first prescription for a TNFI given without there being any prescription for a TNFI in the 2 years before the index date (S1 Fig).

#### Factors for starting biosimilar TNFIs

Baseline demographic and clinical characteristics were collected at the time of starting a TNFI. We used two variables to reflect the economic status of patients. One was the level of their health insurance premium, which reflects annual household income. The other was registration in the individual copayment beneficiaries program for rare and intractable diseases (ICBP), because patients who register in the ICBP program pay only 10% of their medical expenses as a copayment.

Comorbidities were detected for 12 months before the TNFI starting date using the Elixhauser comorbidity index [18]. In addition to the diseases included in that index, we collected information on previous history of special infections such as acute and chronic hepatitis B or hepatitis C, based on ICD 10 codes, as well as on tuberculosis, based on ICD 10 codes, together with use of anti-tuberculosis medication involving at least three of four agents [isoniazid, rifampin (RFP), ethambutol, and pyrazinamide]. A history of treatment for latent tuberculosis was defined as a single use of isoniazid, RFP, or a combination of isoniazid and RFP, before starting a TNFI. Medication with methotrexate, glucocorticoids and nonsteroidal anti-inflammatory drugs (NSAIDs) was identified. To take into account the recommendations for starting TNFI in RA or AS [19, 20], data on medication over a period of 3 months before starting a TNFI were collected to reflect prior disease status.

We also included healthcare utilization factors to account for general patient health, and contact with the healthcare system. These factors included the number of physician visits, number of hospitalizations, and number of emergency room visits in the 1-year prior to starting a TNFI, and the total number of distinct medications dispensed in the 3-month period before starting a TNFI.

### Statistical analysis

We assessed the proportion of each TNFI prescribed between 2004 and 2017 in a descriptive manner. To identify factors for starting biosimilar TNFIs, we selected patients with RA or AS among new starters between 2013 and 2017. In describing the characteristics of patients, categorical variables are presented as frequencies and percentages, and continuous variables as means with standard deviations (SD). A logistic regression model was used to identify factors for starting biosimilar TNFIs. All baseline covariates that we identified were included in each model. Elixhauser score was used to adjust for comorbid conditions. A variance inflation factor of <10 was used as an indication for multicollinearity, and was not observed. The c-statistic was used to compare goodness-of-fit among logistic regression models, with possible values ranging from 0.5 to 1.0 (a value of 0.5 indicates that prediction using the model is no better than chance, whereas a value of 1.0 indicates that a model predicts perfectly).

All analyses were performed with SAS 9.2 (SAS Institute, Cary NC, USA).

### Ethical considerations

This study was determined to be exempt from IRB review by our University Hospital IRB (IRB file No. HYUH 2017-09-033) because we used existing, publicly available data and the subjects could not be identified directly or through identifiers linked to them.

## Results

### Annual changes in the proportions of TNFIs prescribed for RA or AS between 2004 and 2017

**Fig 1:**
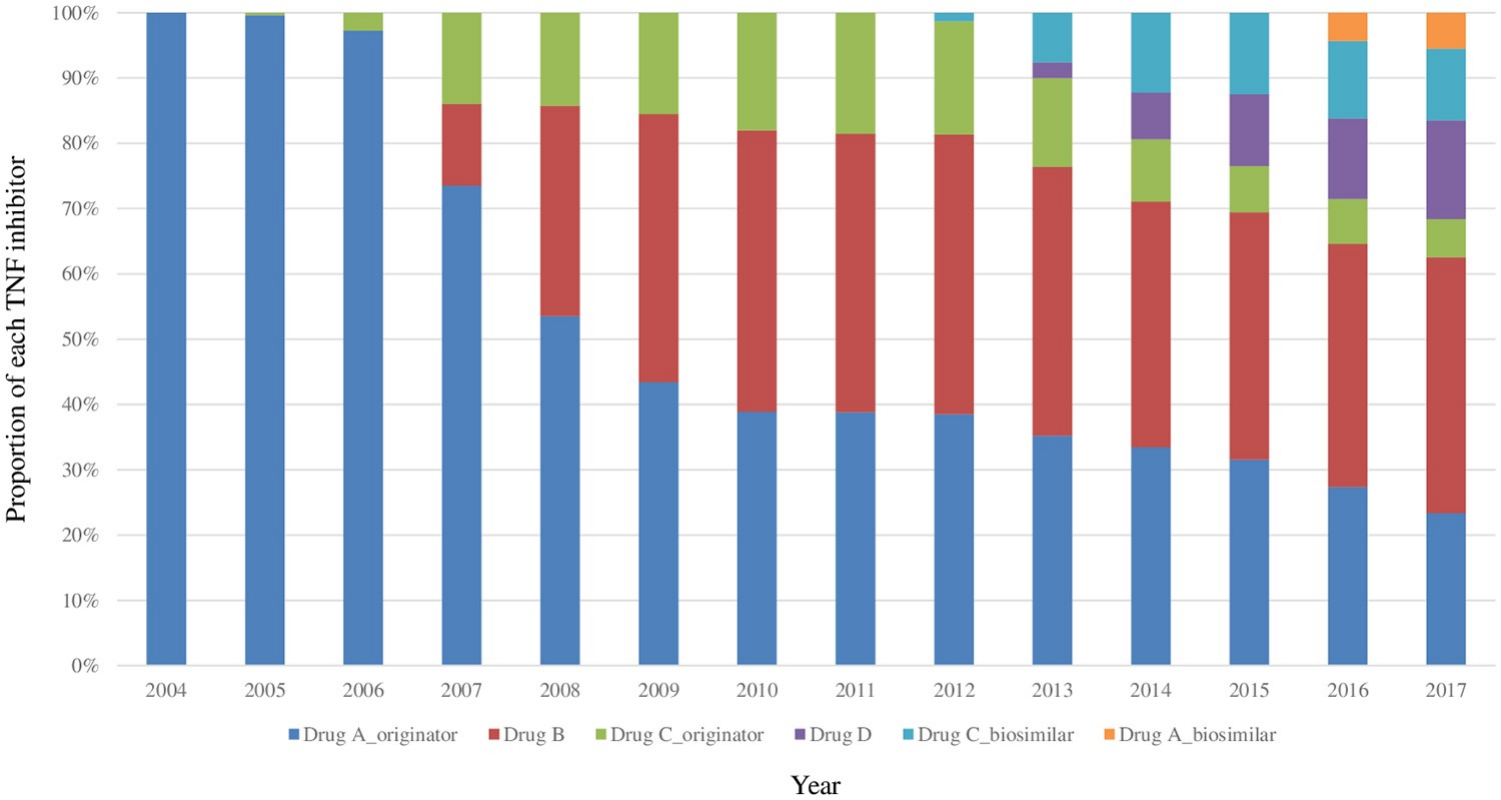
Use of each TNF inhibitor in patients with RA or AS since the approval of TNF inhibitors: Proportions of each TNF inhibitor used by patients with RA or AS since the approval of TNF inhibitors.

After its approval in 2004, Drug A was the most commonly used TNFI until 2009, after which its use decreased gradually with the introduction of other TNFIs such as Drug C in 2005 and Drug B in 2007. The use of all three original TNFIs then decreased gradually with the introduction of biosimilar TNFIs in 2013. The proportion of biosimilar use among all patients prescribed TNFIs increased to 16.5% through 2017 (Fig 1).

**Fig 2:**
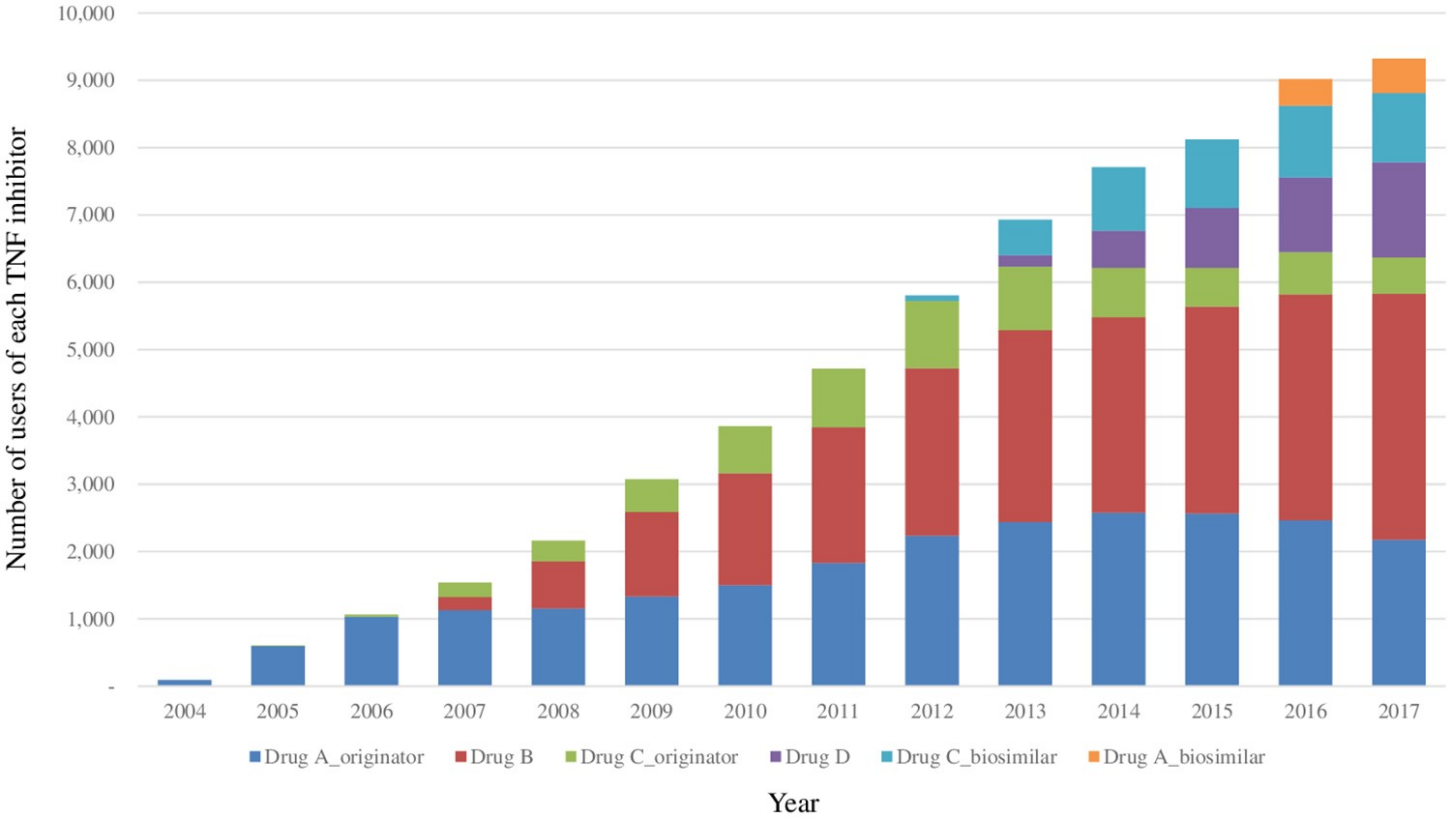
Use of each TNF inhibitor in patients with RA or AS since the approval of TNF inhibitors: Numbers of users of each TNF inhibitor with RA or AS since the approval of TNF inhibitors.

The numbers of patients using each TNFI are presented in Fig 2. After the introduction of the Drug C biosimilar at the end of 2012, the number of Drug C originator users decreased, while the number of Drug A users including both originator and biosimilar and Drug B users increased continuously. After the introduction of the Drug A biosimilar in 2016, Drug A originator users decreased.

### Demographic and clinical characteristics of patients who started TNFIs

We identified a total of 4,216 patients with RA and 2,338 patients with AS who started TNFIs. Table 1 compares the demographic and clinical characteristics of originator users and biosimilar users with RA or AS.

**Table 1:** Demographic and clinical characteristics of RA and AS patients starting the originator and biosimilar TNF inhibitors.

Characteristic	RA			AS		
	Originator starter (n = 3,748)	biosimilar starter (n = 468)	P value	Originator starter (n = 2,023)	biosimilar starter (n = 315)	*P*
Age, mean ± SD	54.5 ± 13.96	54.34 ± 12.8	0.81	39.62 ± 13.69	38.53 ± 13.59	0.19
0–39	557 (14.9)	65 (13.9)	0.04	545 (26.9)	95 (30.2)	0.56
40–49	643 (17.2)	75 (16.0)		528 (26.1)	84 (26.7)	
50–59	1,080 (28.8)	167 (35.7)		464 (22.9)	67 (21.3)	
60–69	972 (25.9)	111 (23.7)		314 (15.5)	40 (12.7)	
70-	496 (13.2)	50 (10.7)		172 (8.5)	29 (9.2)	
Female	2,972 (79.3)	381 (81.4)	0.29	434 (21.5)	74 (23.5)	0.41
Type of insurance						
Medical insurance	3,520 (93.9)	441 (94.2)	0.79	1,930 (95.4)	299 (94.9)	0.71
Medical Aid	228 (6.1)	27 (5.8)		93 (4.6)	16 (5.1)	
Geographic region of patients						
Seoul special city (capital of Korea)	825 (22.0)	69 (14.7)	<0.01	459 (22.7)	78 (24.8)	0.01
Six metropolitan cities*	927 (24.7)	135 (28.9)		496 (24.5)	98 (31.1)	
Other cities/counties	1,996 (53.3)	264 (56.4)		1,068 (52.8)	139 (44.1)	
Household income level for premium						
1–5	638 (17.0)	83 (17.7)	0.67	326 (16.1)	53 (16.8)	0.52
6–10	962 (25.7)	118 (25.2)		551 (27.2)	75 (23.8)	
11–15	884 (23.6)	120 (25.6)		492 (24.3)	86 (27.3)	
16–20	1,264 (33.7)	147 (31.4)		654 (32.3)	101 (32.1)	
Type of institution						
Tertiary hospital	2,402 (64.1)	347 (74.2)		1,302 (64.4)	225 (71.4)	
General hospital	1,076 (28.7)	69 (14.7)	<0.01	549 (27.1)	47 (14.9)	<0.01
Hospital	166 (4.4)	31 (6.6)		152 (7.5)	42 (13.3)	
Clinic/others	104 (2.8)	21 (4.5)		20 (1.0)	1 (0.3)	
Geographic region of institutions						
Seoul special city (capital of Korea)	1,574 (42.0)	141 (30.1)	<0.01	850 (42.0)	130 (41.3)	<0.01
Six metropolitan cities*	1,069 (28.5)	178 (38.0)		582 (28.8)	121 (38.4)	
Other cities/counties	1,105 (29.5)	149 (31.8)		591 (29.2)	64 (20.3)	
Type of department						
Internal medicine	3,483 (92.9)	444 (94.9)	0.29	1,784 (88.2)	310 (98.4)	<0.01
Orthopedics	209 (5.6)	19 (4.1)		191 (9.4)	3 (1.0)	
Others	56 (1.5)	5 (1.1)		48 (2.4)	2 (0.6)	
Year of the prescription of biologics						
2013	712 (19.0)	84 (18.0)	0.48	396 (19.6)	37 (11.8)	<0.01
2014	928 (24.8)	134 (28.6)		367 (18.1)	53 (16.8)	
2015	692 (18.5)	85 (18.2)		394 (19.5)	76 (24.1)	
2016	743 (19.8)	88 (18.8)		417 (20.6)	63 (20.0)	
2017	673 (18.0)	77 (16.5)		449 (22.2)	86 (27.3)	
Registration with the ICBP	3,220 (85.9)	382 (81.6)	0.01	1,895 (93.7)	295 (93.7)	0.99
Comorbidities						
Elixhauser score^†^, mean ± SD	4.66±6.97	3.43±6	<0.01	3.64±5.91	3.7±5.94	0.87
Congestive heart failure	166 (4.4)	10 (2.1)	0.02	31 (1.5)	5 (1.6)	0.81
Cardiac arrhythmias	116 (3.1)	9 (1.9)	0.16	35 (1.7)	11 (3.5)	0.04
Renal failure	72 (1.9)	3 (0.6)	0.05	30 (1.5)	2 (0.6)	0.30
Liver disease	1,157 (30.9)	121 (25.9)	0.03	485 (24.0)	75 (23.8)	0.95
Deficiency anemia	2,023 (54.0)	281 (60.0)	0.01	314 (15.5)	56 (17.8)	0.31
Previous history of special infections^†^						
HBV acute	10 (0.3)	2 (0.4)	0.63	8 (0.4)	1 (0.3)	1.00
HBV chronic	120 (3.2)	16 (3.4)	0.80	40 (2.0)	8 (2.5)	0.51
HCV acute	10 (0.3)	1 (0.2)	1.00	3 (0.2)	1 (0.3)	0.50
HCV chronic	41 (1.1)	3 (0.6)	0.47	15 (0.7)	-	-
Tuberculosis	118 (3.2)	11 (2.4)	0.34	28 (1.4)	2 (0.6)	0.42
History of treatment for latent tuberculosis	880 (23.5)	113 (24.2)	0.75	512 (25.3)	89 (28.3)	0.27
Time to biologics^‡^, mean ± SD (years)	5.57 ± 4.19	5.5 ± 4.14	0.73	3.37 ± 3.81	3.25 ± 3.98	0.60
Number of previous DMARDs, mean ± SD	3.62 ± 1.18	3.6 ± 1.1	0.71	1.12 ± 0.75	1.17 ± 0.75	0.28
Medication^§^						
Methotrexate	3,000 (80.0)	396 (84.6)	0.02	330 (16.3)	48 (15.2)	0.63
Oral glucocorticoids	3,319 (89.0)	402 (85.9)	0.09	920 (45.5)	163 (51.8)	0.04
dose (mg/day, PDS equivalent dose, mean ± SD)	2.58 ± 1.72	2.35 ± 1.61	0.01	2.05 ± 1.73	1.96 ± 1.52	0.53
NSAIDs	3,305 (88.0)	420 (89.7)	0.32	1,879 (92.9)	301 (95.6)	0.08
Healthcare utilization^†^, mean ± SD						
Number of physician visits	39.09 ± 30.15	38.69 ± 29.06	0.78	29.16 ± 26.55	32.81 ± 29.99	0.04
Number of hospitalization	0.93 ± 1.75	1.02 ± 1.53	0.22	0.73 ± 1.58	0.87 ± 1.32	0.09
Number of ER visit	0.42 ± 1.17	0.39 ± 1.24	0.67	0.40 ± 1.38	0.51 ± 2.40	0.45
Number of total distinct medications dispensed	13.68 ± 7.5	14.18 ± 8.0	0.18	9.33 ± 6.39	9.64 ± 6.43	0.43

Characteristics are presented as numbers of patients (%).

*The six metropolitan cities were Busan, Incheon, Daegu, Daejeon, Gwangju, and Ulsan.

^†^Comorbidities and healthcare utilization were analyzed for 365 days before the index date,

^‡^Time to biologics refers to the time between index date and starting a biologic,

^§^Medication was analyzed within 90 days before the index date

RA: rheumatoid arthritis, AS: ankylosing spondylitis, SD: standard deviation, ICBP: the national Individual Copayment Beneficiaries Program, HBV: hepatitis B virus, HCV: hepatitis C virus, DMARD: disease modifying antirheumatic drug, PDS: prednisolone, NSAIDs: nonsteroidal anti-inflammatory drugs

Among the patients with RA, 3,748 originator users and 468 biosimilar users were detected. The mean ages (±SD) of the patients were similar in the two groups (54.5 ± 14.0 years for originator users vs. 54.3 ± 12.8 years for biosimilar users); however, the proportions of younger (≤49 years old) and older (≥60 years old) patients were lower in the biosimilar users than in the originator users (p = 0.04). The proportions of females were similar in the two groups. In terms of geographic regions, the proportions of the patients who lived in the metropolitan area (p<0.01) and attended institutions in the metropolitan area (p<0.01) were higher in the biosimilar users than in the originator users. Registration with ICBP was less frequent among the biosimilar users than the originator users (85.9% vs 81.6%, p = 0.01). Among the patients with AS, we identified 2,023 originator users and 315 biosimilar users. The mean ages and the proportions of females were similar in the two groups. In terms of geographic regions, the proportions of patients who lived in the metropolitan area (p<0.01) and visited institutions in the metropolitan area (p<0.01) were higher among the biosimilar users than among the originator users.

In the RA patients, the Elixhauser comorbidity index was lower in the biosimilar users (3.4±6.0 vs. 4.7±7.0, p<0.01). Concomitant use with methotrexate was higher among the biosimilar users (84.6% vs. 80.0%, p = 0.02), while the mean dose of glucocorticoids (mg/day) was lower (2.4±1.6 vs. 2.6±1.7, p = 0.01). However, in the AS patients, the Elixhauser comorbidity index was similar in the two groups (p = 0.87), as was concomitant methotrexate use (p = 0.63), while concomitant glucocorticoid use was more frequent in the biosimilar users (45.5% in originator users vs. 51.8% in biosimilar users, p = 0.04).

### Factors for starting biosimilar TNFIs

The logistic regression models for factors for starting biosimilar TNFIs yielded c‐statistics of 0.67 in patients with RA and 0.69 in patients with AS.

In patients with RA, biosimilars were more commonly initiated in clinics [odds ratio (OR) 2.54, 95% confidence interval (CI) 1.44–4.47], in institutions in the metropolitan area (OR 2.02, 95% CI 1.47–2.78) and in institutions in other cities (OR 1.82, 95% CI 1.36–2.43), while they were less likely to be initiated in general hospitals (OR 0.40, 95% CI 0.30–0.53) and orthopedics (OR 0.44, 95% CI 0.25–0.79), and in patients registered with the ICBP (OR 0.59, 95% CI 0.44–0.79). In patients with AS, biosimilars were frequently initiated in hospitals (OR 2.20, 95% CI 1.41–3.43), and this use tended to increase over the years (OR 1.16, 95% CI 1.06–1.27), while they were less initiated in orthopedics (OR 0.07, 95% CI 0.02–0.22) and other specialties (OR 0.15, 95% CI 0.03–0.64) (Table 2). In addition, RA patients were more likely to initiate biosimilars in combination with methotrexate (OR 1.37, 95% CI 1.04–1.81), but biosimilars were not initiated for those with higher comorbidity scores (OR 0.97, 95% CI 0.95–0.99) or in combination with glucocorticoids (OR 0.67, 95% CI 0.49–0.91). Patient factors for biosimilar use in AS were not clear.

**Table 2:** Factors for starting biosimilar TNF inhibitor.

Variable	RA*	AS**
Age		
0–39	ref	ref
40–49	0.97 (0.67,1.39)	0.93 (0.66,1.31)
50–59	1.34 (0.97,1.86)	0.88 (0.61,1.26)
60–69	1.08 (0.76,1.54)	0.73 (0.47,1.12)
70-	0.97 (0.63,1.48)	0.98 (0.58,1.67)
Gender		
male	ref	ref
female	1.11 (0.85,1.43)	1.07 (0.79,1.44)
Type of insurance		
Medical Insurance	ref	ref
Medical Aid	0.79 (0.48,1.30)	1.36 (0.70,2.64)
Geographic region of patients		
Seoul special city (capital of Korea)	ref	ref
Six metropolitan cities	1.02 (0.68,1.54)	0.86 (0.55,1.35)
Other cities/counties	1.18 (0.85,1.64)	0.74 (0.52,1.06)
Household income level for premium		
1–5	ref	Ref
6–10	1.00 (0.72,1.38)	0.76 (0.51,1.15)
11–15	1.06 (0.78,1.44)	1.07 (0.73,1.56)
16–20	0.91 (0.68,1.22)	0.90 (0.62,1.31)
Type of institution		
Tertiary hospital	ref	ref
General hospital	0.40 (0.31,0.54)	0.56 (0.39,0.80)
Hospital	1.46 (0.93,2.29)	2.32 (1.48,3.63)
Clinic/others	2.63 (1.49,4.64)	1.21 (0.14,10.44)
Geographic region of institutions		
Seoul special city (capital of Korea)	ref	ref
Six metropolitan cities	1.98 (1.44,2.74)	1.34 (0.91,1.99)
Other cities/counties	1.80 (1.34,2.41)	0.99 (0.67,1.46)
Type of department		
Internal medicine (including rheumatology)	ref	ref
Orthopedics	0.44 (0.24,0.79)	0.06 (0.02,0.21)
Other	0.50 (0.19,1.32)	0.14 (0.03,0.63)
Year of prescription of biologics	0.98 (0.91,1.05)	1.16 (1.06,1.26)
Time to biologics^†^	1.00 (0.97,1.02)	1.00 (0.96,1.04)
Registration with the ICBP	0.61 (0.46,0.82)	0.88 (0.51,1.49)
Elixhauser score	0.97 (0.95,0.98)	0.99 (0.97,1.01)
Number of DMARDs	0.99 (0.90,1.09)	1.01 (0.82,1.25)
MTX	1.36 (1.03,1.81)	0.83 (0.55,1.26)
Oral glucocorticoids	0.65 (0.48,0.88)	1.26 (0.97,1.65)
NSAIDs	1.17 (0.83,1.64)	1.47 (0.81,2.68)
Healthcare utilization		
Number of physician visits	1.00 (1.00,1.00)	1.01 (1.00,1.01)
Number of hospitalizations	1.05 (0.99,1.11)	1.02 (0.95,1.11)
Number of ER visits	0.97 (0.88,1.07)	1.02 (0.95,1.09)
Number of total distinct medications dispensed	1.01 (0.99,1.03)	0.99 (0.97,1.02)

*Hosmer-Lemeshow test, p = 0.54,

**Hosmer-Lemeshow test, p = 0.23

^**†**^Interval between index date and the time of starting a TNF inhibitor

RA: rheumatoid arthritis, AS: ankylosing spondylitis, SD: standard deviation, ICBP: the national Individual Copayment Beneficiaries Program, HBV: hepatitis B virus, HCV: hepatitis C virus, DMARD: disease modifying antirheumatic drug, PDS: prednisolone, NSAIDs: nonsteroidal anti-inflammatory drugs

## Discussion

In Korea, proportional use of biosimilar TNFIs has increased since their introduction. The type of institution and physician specialty influenced the likelihood of starting biosimilars in patients with RA or AS. However, registration with ICBP, high numbers of comorbidities and concomitant medication with glucocorticoids were factors making it less likely for RA patients to start biosimilars. Along with the gradual increase in biosimilar TNFI users, use of the corresponding originator drugs has decreased. However, the consumption of other originator drugs has increased. Accordingly, determining the factors for starting biosimilars rather than originators can provide knowledge about how biosimilars are implemented in the real world. However, to evaluate the impact of biosimilars on other biologic DMARDs, not just TNFI but also all kinds of biologic DMARDs and targeted small molecules will need to be considered.

In our study, type of prescribing institution and department were both factors for starting biosimilars in patients with RA and AS. Thus prescription in hospitals and clinics was a factor for starting biosimilars. This might be related to the fact that more simple administrative procedure is required for drug introduction in hospitals or clinics than in tertiary or general hospitals. Prescription in a department of internal medicine, including a rheumatology department, was a factor for starting biosimilars. Differences in acceptance of biosimilars according to physician specialties have been noted in other studies [21, 22]. They imply that prescription by physicians with specialties that may have more information about, and experience of, biosimilars may be a factor for starting biosimilars. Thus different knowledge or perception on the part of physicians can affect the selection of a biosimilar. The location of an institution in metropolitan areas or other cities compared to the capital was a factor for starting biosimilars in RA patients. Regional differences in choosing biosimilars have been described in another study [21]. Differences in marketing by pharmaceutical companies, as well as differences in the knowledge and opinions of clinicians, may contribute to these regional differences [23].

RA patients who used oral glucocorticoids were less likely to start biosimilars, while those who used MTX were more likely to do so. This may be related to uncertainty about drug effectiveness among both patients and physicians, and is consistent with an observational study using the Korean biologics registry, BIOlogics Pharmacoepidemiologic StudY (BIOPSY) [9]. Further investigation exploring the impact of oral glucocorticoids on starting biosimilars, which could shed more light on our speculation, will be needed. In addition, patients with many comorbidities were less likely to start biosimilars, possibly due to concerns about drug safety in patient and physician. A recent observational data also detected differences in the demographic and clinical characteristics between switchers and non-switchers despite the existence of national guidelines. The clinical decision to switch a patient or not was associated with certain patient characteristics: patients with more comorbidities, higher disease activity and prior failed biological treatments were less likely to be switched. This important finding may reflect uncertainty among patients and rheumatologists on how to implement a newly introduced biosimilar in routine care [12]. On the other hand, in patients with AS, clinical characteristics such as comorbidities or concomitant medications were not associated with starting biosimilars. This may be related to the fewer comorbidities in AS patients, who were younger than RA patients.

RA patients who were registered with the ICBP, which means patients with lower cost sharing, were less likely to select biosimilars. However, household income was not a significant factor for selecting biosimilars. These findings may point to a direct impact of the benefit in terms of cost reduction in patients with rare disease on the choice of expensive drugs rather than of their affordability for medical use. In addition, physicians may take into account patients’ economic status when selecting a biosimilar.

This study has several strong points. First, biosimilars were approved as early as 2012, and the reimbursement guidelines in Korea recommended biosimilars as equal to originators in patients with rheumatic diseases. Therefore, we were able to use long-term observational data to analyze drug utilization tendencies. Second, our data were representative of all patients because we extracted them from a database covering the entire Korean population by stratified sampling by age, gender, and disease. Therefore, our study reflects the selection of drugs by patients and physician in the real world. Third, various economic and regional factors that could affect patients’ and physicians’ choice of drugs were considered. In addition, we examined the impact of acute or chronic comorbidities including hepatitis and latent tuberculosis infection on the selection of drugs.

There are several limitations to this study. First, we could not evaluate clinical effectiveness and safety, because our data did not include results for inflammatory markers or joints counts. Besides cases of switching to biosimilars for economic reason or as a result of government policy, the effectiveness of biosimilars in patients who experienced a lack of efficacy or low tolerability of an originator drug may need to be evaluated. A general lack of knowledge about switching, extrapolation, interchangeability, and overall safety may inhibit the prescription of biosimilars [22]. Therefore, further observational studies, as well as well-controlled clinical trials evaluating the effectiveness, safety and economic benefit of biosimilar use in various patients, are needed to improve our knowledge of biosimilars [24]. Second, we could not examine factors for selecting biosimilars as second biologic DMARDs. Further research evaluating factors related to the selection of biosimilars in various circumstances should provide useful information on how biosimilars are implemented in the real world. Third, a few patients with AS in this study may have taken TNFIs in combination with other rare diseases that have indications for TNFIs. There has been no validation study for identifying AS patients, whereas identifying RA patients having diagnostic codes with prescriptions for biologics or any DMARDs in the Korean claims data was validated in a previous study [25]. Even though most of the AS patients were registered in the ICBP program, some may have had other diseases that were treated with TNFI.

In conclusion, the proportion of biosimilar TNFI use has increased in Korea. The type of institution and the specialty of physicians appear to be more important than patient factors in favoring the choice of biosimilars. In RA, biosimilar TNFIs tend to be used in combination with MTX and are less likely to be initiated by patients receiving glucocorticoids or those with high levels of comorbidities. Thus, factors for starting biosimilars differ in patients with RA versus AS.

## Supporting information

S1 Fig Study participants. (DOCX)

S1 Table The reimbursement approval state of TNF inhibitors in RA and AS in Korea. (DOCX)
