# Supplementary material for: Factors for starting biosimilar TNF inhibitors in patients with rheumatic diseases in the real world
Source: PLoS One. 2020 Jan 24;15(1):e0227960. doi: 10.1371/journal.pone.0227960 (PMC6980538; doi:10.1371/journal.pone.0227960)
Supplement: S1 Fig — (DOCX) [file pone.0227960.s001.docx]

S1 Fig. Study participant

RA or AS patients who used TNF inhibitors between Jan. 2004 and Dec. 2017 (15,124)

TNF inhibitor users with definite RA (n=7,592) or AS (n=4,404) between Jan. 2004 and Dec. 2017

TNF inhibitor starters between Jan. 2013 and Dec. 2017 (RA 4,216, AS 2,338)

Patients with diagnostic codes for both RA and AS (n=3,128)

RA (n=3,376) and AS (n=2,066) patients who started TNF inhibitors between Jan. 2004 and Dec. 2012

1. **Prevalence of the use of TNF inhibitors**
2. **Factors for starting biosimilar TNF inhibitors**
