# Supplementary material for: Factors for starting biosimilar TNF inhibitors in patients with rheumatic diseases in the real world
Source: PLoS One. 2020 Jan 24;15(1):e0227960. doi: 10.1371/journal.pone.0227960 (PMC6980538; doi:10.1371/journal.pone.0227960)
Supplement: S1 Table — (DOCX) [file pone.0227960.s002.docx]

S1 Table. The reimbursement approval state of TNF inhibitors in RA and AS in Korea

|  | RA | AS |
| --- | --- | --- |
| Etanercept | 2004 | 2005 |
| Adalimumab | 2007 | 2007 |
| Infliximab | 2008 | 2012 |
| Golimumab | 2013 | 2013 |
| Infliximab biosimilar A | 2012 | 2012 |
| Infliximab biosimilar B | 2015 | 2015 |
| Etanercept biosimilar | 2015 | 2015 |

RA: rheumatoid arthritis, AS: ankylosing spondylitis
